# Specialist hybrid models with asymmetric training for malaria prevalence prediction

**DOI:** 10.3389/fpubh.2023.1207624

**Published:** 2023-09-21

**Authors:** Thomas Fisher, Sergio Rojas-Galeano, Delmiro Fernandez-Reyes

**Affiliations:** ^1^Department of Computer Science, Faculty of Engineering Sciences, University College London, London, United Kingdom; ^2^Facultad de Ingeniería, Universidad Distrital Francisco José de Caldas, Bogotá, Colombia

**Keywords:** malaria prevalence prediction, ML hybrid models, recurrent network models, asymmetrical loss, machine learning

## Abstract

Malaria is a common and serious disease that primarily affects developing countries and its spread is influenced by a variety of environmental and human behavioral factors; therefore, accurate prevalence prediction has been identified as a critical component of the Global Technical Strategy for Malaria from 2016 to 2030. While traditional differential equation models can perform basic forecasting, supervised machine learning algorithms provide more accurate predictions, as demonstrated by a recent study using an elastic net model (REMPS). Nevertheless, current short-term prediction systems do not achieve the required accuracy levels for routine clinical practice. To improve in this direction, stacked hybrid models have been proposed, in which the outputs of several machine learning models are aggregated by using a meta-learner predictive model. In this paper, we propose an alternative specialist hybrid approach that combines a linear predictive model that specializes in the linear component of the malaria prevalence signal and a recurrent neural network predictive model that specializes in the non-linear residuals of the linear prediction, trained with a novel asymmetric loss. Our findings show that the specialist hybrid approach outperforms the current state-of-the-art stacked models on an open-source dataset containing 22 years of malaria prevalence data from the city of Ibadan in southwest Nigeria. The specialist hybrid approach is a promising alternative to current prediction methods, as well as a tool to improve decision-making and resource allocation for malaria control in high-risk countries.

## 1. Introduction

Malaria is a severe and widespread disease that affects millions of people worldwide. It is a major public health issue in many developing countries, particularly in sub-Saharan Africa, where the disease burden is highest. Accurate and affordable prediction of malaria prevalence is critical for effective prevention and control measures, as it has been outlined in the 2016–2030 Global Technical Strategy for Malaria (3). Low-cost predictive systems that can provide accurate short-term predictions of malaria prevalence are needed to facilitate informed decision-making and efficient allocation of limited anti-malarial resources in countries at higher risk.

A wide variety of approaches have been employed by researchers to forecast the prevalence of malaria. These methods can be broadly classified into three main categories: mathematical models such as the Susceptible-Infected-Recovered (SIR) model (4), classical data-driven models like Autoregressive Integrated Moving Average (ARIMA), and, more recently, Machine Learning models (ML) such as Elastic Net and Neural Networks.

Prediction systems have traditionally relied on classical differential equation models (e.g., SIR) of transmission dynamics. Such models can predict basic forecasts based on explicit biological assumptions about disease communication. Malaria prevalence, however, is a complex variable influenced by a variety of environmental and human behavioral factors and as a result, data-driven ML techniques that incorporate these and other clinical factors can outperform explicit models in terms of accuracy. Supervised ML algorithms, in particular, can predict future prevalence levels without relying on predefined assumptions, which are often too simplistic to capture the complexity of real-world phenomena. Hence, data-driven ML techniques have emerged recently as a more reliable approach for malaria prevalence prediction (5).

For instance, Brown et al. (5) conducted an investigation utilizing a regional dataset of malarial occurrence in over 90,000 individuals from the city of Ibadan in southwest Nigeria, across 22 years. Employing an Elastic Net Model, dubbed REMPS (Region-specific Elastic-net based Malaria Prediction System), the authors were able to predict malaria prevalence 1-month in advance. This particular ML approach was found to outperform alternative methods such as LASSO, Ridge Regression, Least Angles Regression, Random Forest, and Support Vector Regression approaches. By demonstrating adequate predictive performance within a specified problem-specific error tolerance, that study highlighted the potential advantages of ML approaches in modeling malarial prevalence time series data.

Furthermore, stacked hybrid methods (6, 7), involve the aggregation of predictions generated by various machine learning and classical models via a meta-learner, such as a weighted voting, to produce a final output prediction. This combination of diverse models is an extension of the ensemble method (10). The stacked hybrid of neural networks and exponential smoothing models, proposed by (6), was highly successful in the M4 time series forecasting competition (8), which included 10,000 different datasets from a variety of sources. Atiya (9) carried out a thorough examination of the stacked hybrid model, which outperformed many other complex methods and Zhang (11) demonstrated the power of specialist hybrid models which are outperform the more complex model components on a range of real-world datasets. This clearly demonstrates the potential of hybrid models for timeseries forecasting problems.

In the existing literature on malaria prevalence prediction, we came across only one study that utilized a stacked hybrid model (2). This approach employed gradient boosted regression trees (GBRTs) as meta-learners to enhance predictive performance by combining autoregressive, neural network, and Long-Short Term Memory (LSTM) predictors. However, specialist hybrid models tailored to address the malaria prevalence prediction problem have not been explored in prior works. Thus, our contribution in this paper is to bridge this gap in the literature by introducing and deploying specialist hybrid models to this domain. Our results highlight the effectiveness of these models compared to other ensembling and stacked hybrid approaches, offering valuable insights for improved predictive performance.

## 2. Materials and methods

### 2.1. Dataset and preprocessing

The dataset employed in this study (1), was previously introduced by (5). It comprises monthly observations of the variables described in Table 1 and spans the period from January 1996 to December 2017, inclusive, gathered from Ibadan, Nigeria.

**Table 1:** Description of monthly variables available in our working dataset. MPs/μl stands for malaria parasites per microliter.

**Notation**	**Variable**	**Description**
*t*	month	Number of months from the start of the observation period
*I*_*t*_	intervention	Anti-malarial interventions in place (categorical)
*X*_*t*_	Median-age-neg	Median age (months) of children who tested malaria-negative
	median-age-pos	Median age (months) of children who tested malaria-positive
	iqr-age-neg	IQR of the age (months) of children who tested malaria-negative
	iqr-age-pos	IQR of the age (months) of children who tested malaria-positive
	x-pd	Mean blood-parasite density (MPs/μl)
	sd-pd	Standard deviation of blood-parasite density (MPs/μl)
	mm-rf	Rainfall (mm)
	min-temp	Minimum temperature (°C)
	max-temp	Maximum temperature (°C)
	x-temp	Mean temperature (°C)
*y*_*t*_	prevalence	Proportion of those screened with confirmed malaria

Our target variable is the malarial prevalence which represents the proportion of the population that tests positive for malaria during a given month. We denote this variable as *y*_*t*_, where *t* represents the month. In addition to historical prevalence values, our models incorporate environmental and clinical observation values as predictor variables. We represent the vector of these observations as *X*_*t*_. Moreover, the anti-malarial intervention category in place during month *t* is denoted as *I*_*t*_. Table 1 summarizes the notation and description for these variables.

Regarding the categorical variable *I*_*t*_, Table 2 summarizes the intervention categories during the observation period. The “+” symbol indicates interventions deployed on top of preceding ones. For instance, months corresponding to intervention category +IRS-Rec have ITN-Free, IPT-Preg, and IRS-Rec interventions in place. Note that both ITN-All and ACT-Free interventions were introduced simultaneously, so we grouped them together due to the lack of data from separate deployment periods.

**Table 2:** Intervention categories used for variable *I*_*t*_.

***I*_*t*_**	**Intervention**	**Explanation**
0	None	No interventions in place
1	ITN-Free	Free insecticide-treated bed nets are given to children
2	+IPT-Preg	Intermittent preventative therapy is given for free to pregnant women
3	+IRS-Rec	Indoor residual spraying is recommended
4	+ITN-All and ACT-Free	ree insecticide-treated bed nets are given to everyone and free artemisinin-based combination therapy is available
5	+Larval-Cont	Larval control measures in mosquito breeding grounds

### 2.2. Forecasting lead length and forecasting window

We focus on short-term predictions for a 1-month horizon as the forecasting lead length. Such an approach is pervasive in the literature on forecasting. We adopt a forecasting window of 3 months, which enables models to access information from a local (temporal) neighborhood. This feature is particularly relevant for sequential models such as Long Short-Term Memory (LSTM) networks to facilitate effective learning. Furthermore, we purposefully omitted the calendar month information from our training procedures to enable the system to respond to variations in the malaria season that may arise due to changes in the global climate.

### 2.3. Train-test split and cross validation

The dataset contains 264 months' worth of recorded observations. We split the dataset into two parts: a training set with the first 190 months of data and a test set with the remaining 74 months of data. The test set was held-out as unseen data to assess our models' ability to generalize to future forecasts. We used five-fold cross-validation during the training stage of our models.

### 2.4. Measures of performance

We will initially train our models using MSE (Mean Squared Error) minimization, but we will also evaluate their quality using the MPET (Malarial Prevalence Error Tolerance) measure introduced in (5). The MPET is calculated as follows:


(1) $$\begin{array}{l} \text{MPET}:=\frac{100}{n}\sum_{k=1}^{n}1[-0.05\leq ({\hat{y}}_{k}-y_{k})\leq 0.1] \end{array}$$

where **1**[·] is an indicator function that maps to either 0 or 1 if the prediction falls within the specified condition interval, *n* refers to the size of the test set being evaluated, and *y*_*k*_ and ŷ_*k*_ represent the true observed and predicted values at time *k*, respectively. The MPET ranges between 0 and 100%, and both the MPET and MSE are valuable metrics for ranking the predictive performance of our models. More accurate models will exhibit smaller MSE and larger MPET scores compared to weaker models.

It should be noted that the MPET is a metric that is more tailored to specific applications due to its preference for overprediction. This preference stems from the fact that decision-makers often choose to base their decisions on more pessimistic estimates rather than optimistic ones. In this context, it is preferable to overestimate rather than underestimate the prevalence of malaria.

### 2.5. Preprocessing

To prevent large error gradients from causing instability in the learning process, we scaled each continuous variable's observations between the minimum and maximum observed values. This helps ensure that weight values do not fluctuate wildly during training, particularly for gradient-based methods used in this investigation.

Our findings indicate that superior predictive performance can be achieved by training models on the transformed dataset described in the Supplementary material. Specifically, we observed that when models were trained on the transformed data, their MSE values were roughly halved and their MPET values increased by around 10% on average, compared to training regimes using the original data.

### 2.6. Baseline models

We applied a variety of classical and machine learning time series forecasting methods to the dataset. Full implementation details can be found in the Supplementary material. The classical models used were Holt-Winters (also known as Triple Exponential Smoothing) and SARIMAX (Seasonal Auto-Regressive Integrated Moving Average with Exogenous variables). To train the classical models, we utilized an autoregressive approach with a 3-month forecasting window, using the malaria prevalence signal {*y*_*t* − 3_, *y*_*t* − 2_, *y*_*t* − 1_}. The SARIMAX model included the malaria prevalence signal as well as the intervention used during those months {*I*_*t* − 3_, *I*_*t* − 2_, *I*_*t* − 1_} as an exogenous variable.

The ML models we implemented were EN (Elastic Net), RF (Random Forest), SVR (Support Vector Regression), NN (Neural Network), and BiLSTM (Bidirectional Long Short-Term Memory). In addition to the malaria prevalence signal and the intervention, the ML models were also trained on the past three months of environmental and clinical observations {*X*_*t* − 3_, *X*_*t* − 2_, *X*_*t* − 1_}. To ensure more reliable estimates, we trained 10 different instances of the best-performing deep network models (i.e., NN and BiLSTM) based on validation loss, and report the average results.

Details regarding the hyperparameter selection for these baseline models can be found in the Supplementary material.

#### 2.6.1. SARIMAX

Using the Akaike Information Criterion (AIC) (12), we identified the optimal parameter set for our SARIMAX model to be (*p, d, q, P, D, Q, S*) = (2, 0, 3, 2, 0, 3, 12). This resulted in an AIC of −330.842 when the intervention was considered as a categorical exogenous variable. The seasonality order *S* was set to 12 since we know that the data has a seasonal period of 12 months due to the mosquito lifecycle. This was the only way in which temporal information from outside of the 3-month forecasting lead window was provided to the autoregressive models. The remaining order parameters were selected from a range of 0, …, 6, and a grid search was conducted to determine their optimal values. For more information about the SARIMAX model, please refer to the Supplementary material.

#### 2.6.2. BiLSTM

**Figure 1:**
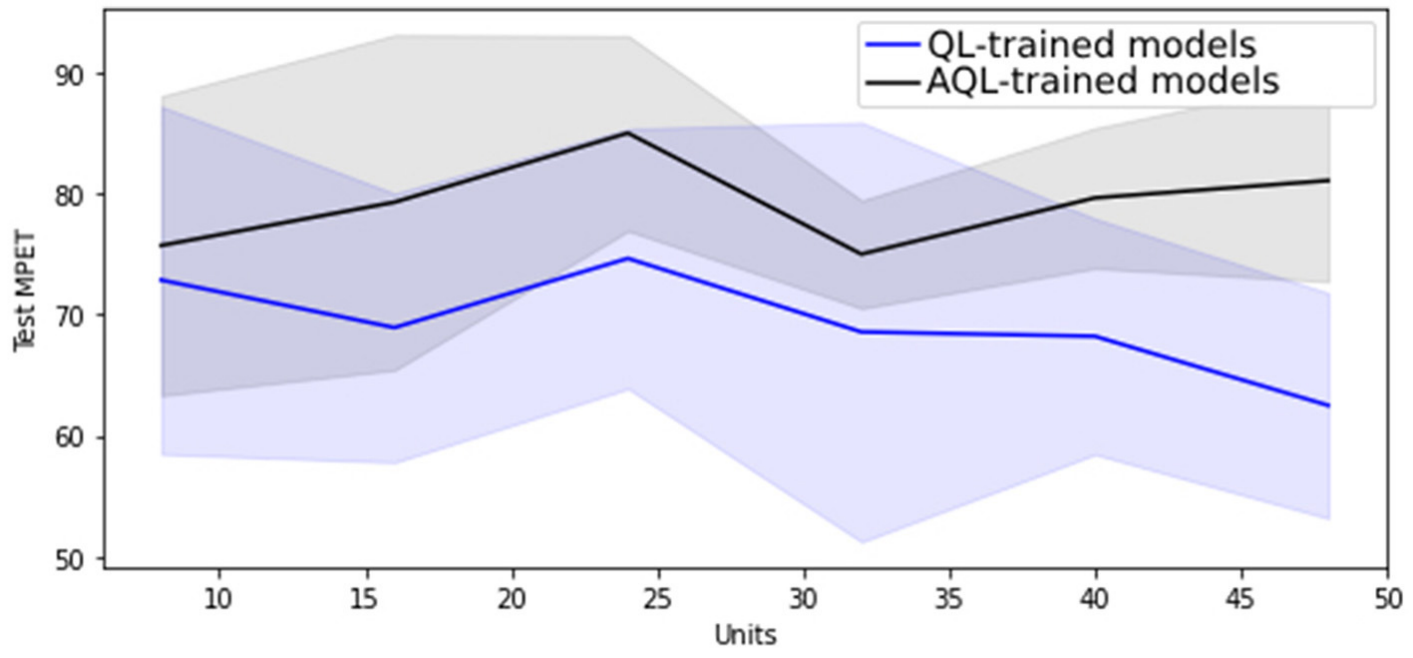
Mean MSE scores with standard deviation as shaded area for 20 randomly initialized BiLSTM models of differing sizes trained with symmetric and asymmetric quadratic losses.

**Figure 2:**
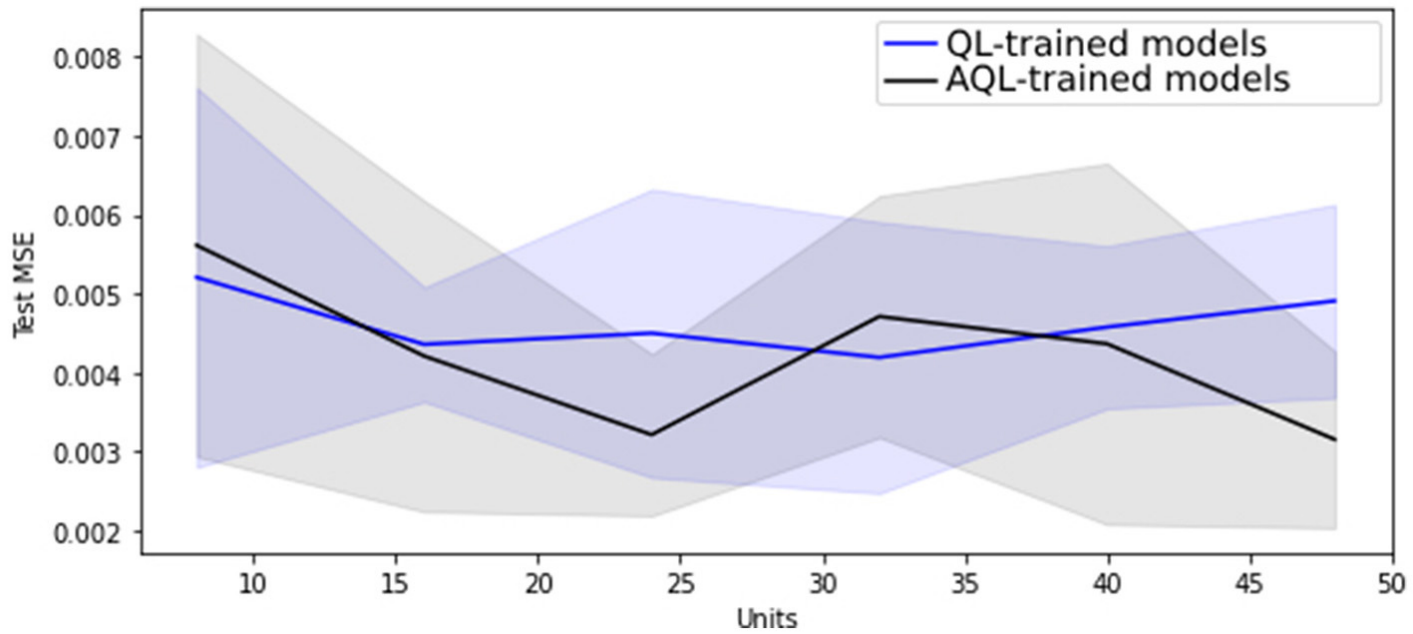
Mean MPET scores with standard deviation as shaded area for 20 randomly initialized BiLSTM models of differing sizes trained with symmetric and asymmetric quadratic losses.

Regarding hyper-parameter tuning of our models, we chose a 24-unit Bidirectional Long Short-Term Memory (BiLSTM) architecture based on its superior performance, as demonstrated in Figures 1, 2, showing the lowest validation set MSE loss and highest MPET scores, respectively. We utilized the Adam optimizer (13) and conducted training for 100 epochs with a batch size of 30. For further information on LSTM models, please refer to the Supplementary material.

### 2.7. Weighted ensemble methods

We created ensembles by training 20 separately initialized models and weighting them based on their performance on the validation set. Validation sets were chosen randomly as held-out subsets of the training data of size 20%. We calculated the weights according to Equation (2):


(2) $$\begin{array}{ll} w_{i} & =\frac{e^{-s_{i}}}{\sum_{i}e^{-s_{i}}} \end{array}$$

Here, $s_{i}=\frac{v_{i}}{\sum_{i}v_{i}}$ represents a normalized score obtained from validation set MSE *v*_*i*_ achieved by model *i*. In this way, higher weights are assigned to models with better validation scores, making them more influential in the ensemble.

This means that the output prediction ${\hat{N}}_{t}^{E}$ from a weighted ensemble *E* of *K* non-linear models *N*_*k, t*_ for *k* ∈ 1, …, *K*, at time *t* is


(3) $$\begin{array}{ll} {\hat{N}}_{t}^{E} & :=\overset{K}{\underset{k=1}{\sum }}w_{k}{\hat{N}}_{k,t} \end{array}$$

### 2.8. Stacked hybrid models

As an alternative to ensembles, Wang et al. (2) trained a variety of classical and neural forecasters and then combined them with Gradient Boosted Regression Trees (GBRT) as meta-learners. We implemented their method, but provided the neural forecasters with both environmental and clinical data, rather than just the environmental data that they used. For our stack of models, we chose our best performing classical and neural models: SARIMAX and BiLSTM, respectively. We implemented the GBRT technique using the Python sklearn library with default hyperparameters (MSE loss, learning_rate=0.1, and n_estimators=100), as the study (2) did not specify on this regard.

### 2.9. Asymmetric quadratic loss (AQL)

The previous methods applied to this dataset (5) utilized the MPET metric to evaluate the success of a monthly prediction. However, in order to use this metric in training through backpropagation, a differentiable loss function is required. When training with a differentiable version of the MPET loss, the model's performance is adversely affected due to the “bucket” shape of the loss function, which causes gradients to become very small unless evaluated at the specific values which are the “walls” of the bucket.

We have found that using a symmetric quadratic loss *QL*(*y*, ŷ) = (*y* − ŷ)^2^ during training is much more advantageous as it consistently reduces both the MSE and MPET metrics simultaneously. However, since MPET is an asymmetric metric that penalizes models more heavily for underestimating the true value than for overestimating it, it is inappropriate for our purpose to use a symmetric quadratic loss that penalizes both over and underestimation equally. We need a loss function that improves both MSE and MPET metrics while maintaining a preference for overestimation. To address this issue, we explore the use of an asymmetric quadratic loss *AQL* which is defined as follows:


(4) $$\begin{array}{l} AQL(y,\hat{y};\tau ):=\{\begin{array}{ll} \tau {\left(y-\hat{y}\right)}^{2}, & \text{if }y\leq \hat{y}\\ (1-\tau ){\left(y-\hat{y}\right)}^{2}, & \text{if }y>\hat{y} \end{array} \end{array}$$

The MPET metric penalizes underestimation twice as severely as overestimation, with 0.05 (the size of the lower error tolerance band in Equation 1) being half the size of 0.1 (the size of the upper error tolerance band in Equation 1). To maintain this 2:1 weighting ratio, we select $\tau =\frac{1}{3}$, resulting in twice the penalty for underestimation compared to overestimation.

### 2.10. Specialist hybrid models

The idea of specialist hybrid models (11) is to decompose the true signal *y*_*t*_ into a linear autocorrelation component $y_{t}^{L}$ and a non-linear component $y_{t}^{N}$, i.e., $y_{t}=y_{t}^{L}+y_{t}^{N}$. A linear estimator ${\hat{y}}_{t}^{L}$ is fit on *y*_*t*_ to estimate $y_{t}^{L}$, and then the non-linear residuals $e_{t}=y_{t}-{\hat{y}}_{t}^{L}$ are assumed to be modeled from past observations *e*_*t*_ = *f*(*e*_*t* − 1_, *e*_*t* − 2_, …, *e*_*t* − *n*_) + ϵ_*t*_, where ϵ_*t*_ is the random process noise. A non-linear estimator ${\hat{y}}_{t}^{N}$ is then fit on *e*_*t*_ to estimate $y_{t}^{N}$. The final prediction is obtained by summing the linear and non-linear components:


(5) $$\begin{array}{l} {\hat{y}}_{t}={\hat{y}}_{t}^{L}+{\hat{y}}_{t}^{N} \end{array}$$

This approach allows each component model to specialize in modeling the type of data on which it performs best. By using a non-linear specialist model, the hybrid approach can improve on classical methods that may have difficulty capturing non-linearities in the data.

In this study, we propose to use a SARIMAX model as the linear estimator and a bidirectional long short-term memory (BiLSTM) recurrent neural network as the non-linear estimator. The different models are fitted using distinct data sources. The SARIMAX model uses the prevalence values from the previous 3 months {*y*_*t* − 3_, *y*_*t* − 2_, *y*_*t* − 1_} and the corresponding intervention measures {*I*_*t* − 3_, *I*_*t* − 2_, *I*_*t* − 1_}, as the exogenous variable, to generate the linear model prediction ${\hat{y}}_{t}^{L}$ for the prevalence in month *t*.

The BiLSTM model is fitted using environmental and clinical data {*X*_*t* − 3_, *X*_*t* − 2_, *X*_*t* − 1_} from the prior three months as inputs to predict ${\hat{y}}_{t}^{N}$, the non-linear residuals of the linear model. The approach trains the non-linear model using residuals from the linear prevalence predictions only, whereas our approach uses clinical and environmental data {*X*} instead of residuals (11). Furthermore, we trained the BiLSTM using AQL to encourage a preference for overprediction. The final predictions are obtained by adding the forecasts from the two specialist models, using Equation (5).

The motivation behind our method is that it leverages the unique strengths of the individual models rather than forcing them to perform the same task. We fit our best linear and non-linear models to capture the linear and non-linear components of the signal, respectively. Specifically, our SARIMAX model is trained to predict the linear component, while an ensemble of asymmetrically trained 24-unit BiLSTMs is used to predict the non-linear component. The algorithm for training and prediction of our specialist hybrid model is presented in Algorithm 1.

**Algorithm 1:**
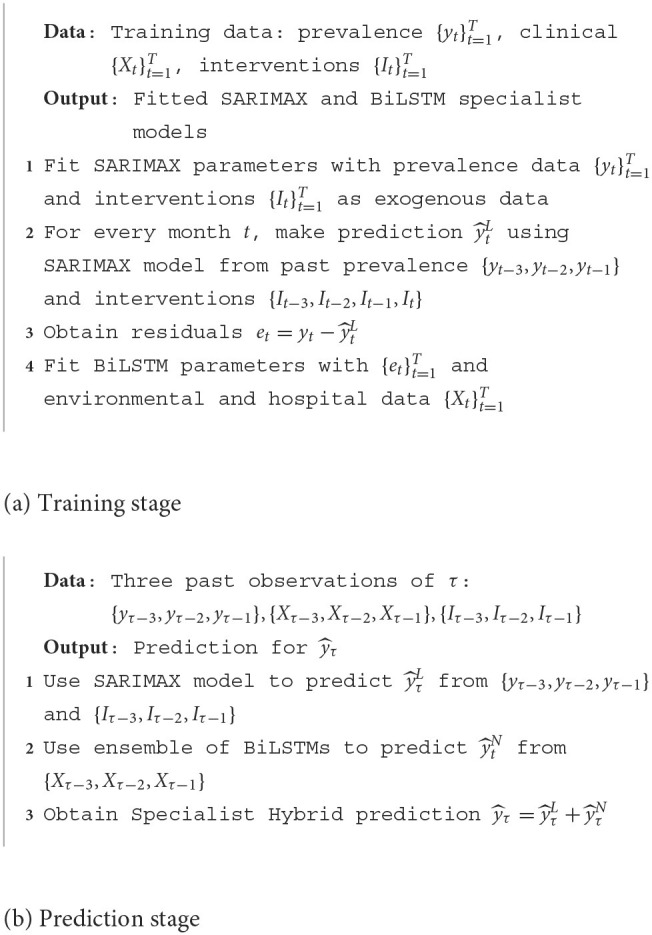
Forecasting malaria prevalence with our proposed specialist hybrid model.

### 2.11. Model predictive variance

In addition to comparing model performance on held-out test sets using MSE and MPET, we are also interested in how much variance there is in the predictions made by the different models. This gives us some idea of certainty of the models in their predictions.

Below we show calculations to estimate the variance made by an ensemble of *K* specialist hybrid models upon presentation of point-wise inputs *x*. Each predictor *k* in the ensemble consists of linear and non-linear component models, denoted by *L*_*k*_ and *N*_*k*_, respectively, *k* ∈ 1, …, *K*. The prediction of the *k*th specialist model in the ensemble is given by ${\hat{L}}_{k}\left(x\right)+{\hat{N}}_{k}\left(x\right)$, where ${\hat{y}}_{k}^{L}:={\hat{L}}_{k}\left(x\right)$ and ${\hat{y}}_{k}^{N}:={\hat{N}}_{k}\left(x\right)$ are the linear and non-linear components' predictions, respectively (here subscripts refer to elements in the ensemble, not to the samples in the data series). The weight given to the *k*th specialist couple in the ensemble is denoted by *w*_*k*_, and the empirical mean over all the predictions in the ensemble is denoted by an overbar. Hence, the variance in overall prediction for input variable *x*, denoted by ν(*x*), is given by the following equation:


(6) $$\begin{array}{l} \nu (x):=\sum_{k=1}^{K}w_{k}{\left({\hat{L}}_{k}(x)+{\hat{N}}_{k}(x)-\bar{\left[{\hat{L}}_{k}(x)+{\hat{N}}_{k}(x)\right]}\right)}^{2}\\ \;=\sum_{k=1}^{K}w_{k}{\left({\hat{N}}_{k}(x)-\bar{{\hat{N}}_{k}(x)}\right)}^{2} \end{array}$$

The reason for this result is that a single linear component is trained and shared among all specialist models in the ensemble. As a result, the linear prediction ${\hat{L}}_{k}\left(x\right)$ is the same for all models in the ensemble, and therefore it cancels out when subtracted from the average ensemble linear component prediction. Therefore, the variance calculation only takes into account the differences between the non-linear component predictions across the ensemble and its average non-linear prediction.

To estimate the total variance across the entire feature space, we integrate the sum of variances for each point-wise prediction:


(7) $$\begin{array}{l} \int dp(x)\sum_{k=1}^{K}w_{k}{\left({\hat{N}}_{k}(x)-\bar{{\hat{N}}_{k}(x)}\right)}^{2}\\ \;\approx \frac{1}{N}\sum_{n=1}^{N}\sum_{k=1}^{K}w_{k}{\left({\hat{N}}_{k}(x_{n})-\bar{{\hat{N}}_{k}(x_{n})}\right)}^{2}\\ \;=:{\bar{\nu }}_{X} \end{array}$$

where *p*(*x*) is the theoretical underlying probability distribution associated with the feature space. To approximate the integral, the sum of variances is averaged over a representative sample of the feature space. This sample is a test dataset $X={x_{n}}_{n=1}^{N}$ with *N* points. The resulting average measure is denoted by ${\bar{\nu }}_{X}$, which is an estimation of the variance of the models' predictions. This estimation assumes that the test data is a valid representative sample of the prevalence signal.

## 3. Results

### 3.1. Performance of baseline models in malaria prevalence prediction

Table 3 presents the average results for prevalence prediction using the MSE and MPET measures of performance with the transformed dataset. It can be observed that the SARIMAX model performs the best among classical models, as it significantly outperforms the Holt-Winters (HW) model with an average of 14.7% lower MSE score and 22.1% higher MPET score.

**Table 3:** Test set performance of models trained on the transformed dataset.

**Model**	**MSE**	**MPET(%)**
HW	0.0068	54.3
SARIMAX	0.0058	76.4
EN	0.0028	77.1
RF	0.0034	80.0
SVR	0.0032	71.4
NN	0.0027	84.7
BiLSTM	0.0024	85.3
Wang et al. (2): GBRT meta-learned SARIMAX BiLSTM stack	0.0022	87.6

Furthermore, Table 3 clearly demonstrates that the BiLSTM model (without ensembling) outperforms the other machine learning models, achieving the lowest MSE of 0.0024 and the highest MPET of 85.3%. The Neural Network (NN) model obtains the second-best performance, with superior MSE and MPET scores compared to the Elastic Net (EN), Random Forest (RF), and Support Vector Regression (SVR) models.

Our findings are in line with those of (2), who proposed that LSTM models are appropriate for this domain. However, we validated this on a much larger dataset than the one used in that study. Moreover, while (2) only investigated standard LSTMs, we discovered that better performance can be obtained by using BiLSTMs, as shown in the Supplementary material. These results confirm our choice to utilize BiLSTM as our base ML model for our specialist hybrid models.

Figure 1 shows that training BiLSTM models of various sizes using the asymmetric AQL instead of the symmetric QL does not have a significant impact on the mean and standard deviation of the MSE performance. However, it also demonstrates that training with AQL instead of QL consistently reduces the mean and standard deviation of the MPET measure of performance across BiLSTM models of various sizes.

The results of using a weighted ensemble of BiLSTM models are presented in Table 4. We can observe that compared to the results obtained using single BiLSTM models in Table 3, there is a 0.0002 and 2.3% improvement in MSE and MPET, respectively, when training with the symmetric MSE loss, which is expected as ensembling typically improves single-based predictive performance. Moreover, we found that using AQL can further improve MPET scores by 5.9% relative to the single BiLSTM model.

**Table 4:** Test set performance of models trained using symmetric (QL) and asymmetric (AQL) quadratic losses. Note that pure SARIMAX method is independent of loss and so the values in both columns are duplicated.

**Model**	**QL**		**AQL**	
	**MSE**	**MPET (%)**	**MSE**	**MPET (%)**
SARIMAX	0.0057	76.3	0.0057	76.3
BiLSTM Ensemble	0.0022	88.6	0.0022	91.2
Our implementation of (2): *Stacked* Hybrid of SARIMAX and BiLSTMs with GBRT Meta-Learner	0.0022	87.6	0.0021	89.4
Our *Specialist* Hybrid of SARIMAX and BiLSTMs	0.0019	91.4	0.0018	97.1

### 3.2. Specialist hybrid models outperform stacked hybrid model in malaria prevalence prediction

We found that our implementation of the stacked hybrid model proposed by Wang et al. (2) achieves remarkable performance, as shown in Table 4. The model obtains an MSE score of 0.0022 and an MPET score of 87.6%, outperforming any of the other baseline models in Table 3 as well as the BiLSTM ensemble in Table 3. Besides, note that this model was trained without asymmetric loss. Based on these results, we establish that the stacked hybrid model is the current state-of-the-art in malaria prevalence prediction with our transformed dataset (2). Therefore, we will use it as a benchmark to evaluate the specialist hybrid model, trained with the alternative AQL loss, that we propose in this study.

Table 4 shows that our specialist hybrid model achieves a lower MSE of 0.0018 and a higher MPET of 97.1% when the BiLSTM component is trained using the proposed AQL loss. These values demonstrate better performance than the MSE and MPET scores of the (2) stacked hybrid approach, which were 0.0021 and 89.4%, respectively, and were also trained with the AQL loss this time.

Table 4 also demonstrates the improvement in performance resulting from training the models using AQL. As expected from our investigation in Figures 1, 2, the MSE values are not noticeably affected by using this loss. However, the MPET scores demonstrate an average improvement of 4% compared to the models trained with the symmetric loss. It is worth noting that these results confirm the effectiveness of the proposed AQL loss in enhancing the predictive performance of the specialist hybrid model.

### 3.3. Model predictive variance comparison

Table 5 presents the values of the total model variance ${\bar{\nu }}_{X}$ as computed by Equation (7). The results indicate that the specialist hybrid model has a significantly lower prediction variance than the non-hybrid BiLSTM ensemble. In both approaches, we used ensembles of size 50.

**Table 5:** Model variance comparison for ensemble and specialist hybrid models.

**Model**	** ${\bar{\nu }}_{X}$ **
BiLSTM	5.5 × 10^ − 5^
Specialist hybrid SARIMAX-BiLSTM	2.3 × 10^ − 5^

## 4. Discussion

Combining the SARIMAX and BiLSTM together in a specialist hybrid model leverages the available data effectively by allowing each component to specialize in modeling the type of data on which it performs best. The SARIMAX model operates on only the prevalence and intervention data as an exogenous variable, whilst the neural system models the non-linear component, the residuals from the SARIMAX model, as a function of the clinical and environmental data.

Machine learning models can effectively predict malaria prevalence by using clinical and environmental data, although they require a larger amount of data for parameter tuning and training, which makes the process computationally expensive. In contrast, the traditional linear component of the specialist hybrid model is more robust and has fewer parameters to tune, reducing the risk of overfitting to training data. By using the specialist hybrid model, the high-variance non-linear BiLSTM model can predict on a smaller portion of the total prevalence while the SARIMAX model can handle the linear or autoregressive component of the signal. The neural non-linear model can be interpreted as fine-tuning the classical model, resulting in consistently higher accuracy, as other studies have also found (11).

In terms of the bias-variance tradeoff, specialist hybrid models do not significantly increase the bias as the non-linear neural model can still model a substantial part of the signal, and the linear component has fewer parameters to tune. The hybrid model provides a way to effectively combine the strengths of both linear and non-linear models while mitigating their weaknesses. Therefore, specialist hybrid models can be applied to other real-world applications with little disadvantage, and we advocate for their more widespread deployment.

Real-world datasets are often small, making it challenging to train neural systems that typically require larger sample sizes to generalize well. By reducing the total model variance ${\bar{\nu }}_{X}$ to less than half of that of the non-hybrid model as shown in Table 5, the hybrid approach more effectively addresses overfitting issues.

Our specialized hybrid approach is novel in the malaria prevalence prediction literature and advantageous to the other hybrid approaches taken in this area. Compared to (2), who actually work on the same problem, we achieve comprehensively better performance and from a theoretical standpoint our approach only uses the neural models (which have higher predictive variance) on a smaller (non-linear) part of the signal, meaning that the total hybrid model predictive variance is lower. The approach by (11) has not been applied to this area but is less useful for malarial prevalence prediction as it only uses residuals from the linear model as input to the non-linear model, whereas in this application we have access to clinical and environmental covariates which we know impact the signal and which we use in our approach to model the non-linear deviation from the overall linear autoregressive trend.

As an additional remark, the use of the newly proposed asymmetric training approach with AQL yielded improved performance across all models. Notably, the MPET scores specific to and very important for malaria prevalence prediction problem, in particular, exhibited a noticeable improvement with the use of this loss function.

## 5. Conclusion

In this paper, we introduced new methodology to the malaria prevalence prediction problem in the form of asymmetric training and specialist hybrid model approaches. Our findings suggest that both the specialist hybrid approach and the use of the asymmetric quadratic loss are promising methodologies individually for malaria prevalence prediction. Combining the two into an asymmetrically trained specialist hybrid model outperformed the other methods in the literature, including state-of-the-art stacked hybrid models. The specialist hybrid approach is therefore a promising alternative to current prediction methods, and should be deployed by local stakeholders to improve decision-making and resource allocation for malaria control in high-risk regions.

We recognize a limitation in our research pertaining to the dataset's geographical origin, which is confined to the Ibadan region in Nigeria. Consequently, the generalizability of our findings might be limited. To address this concern, we intend to extend our model's application to diverse regions worldwide, where malaria displays varying seasonality patterns influenced by distinct weather conditions (15).

Expanding our deployment to different regions will enable us to assess the model's stability, explore variations in optimal hyperparameters, and provide valuable insights for the model retraining pipeline in clinical practice. Furthermore, incorporating a broader range of variables that account for regional mechanisms (14), including a wider range of environmental factors, demographic characteristics such as age groups, and clinical information such as comorbidities, may enhance the accuracy and robustness of our specialist hybrid approach.

## Data availability statement

Publicly available datasets were analyzed in this study. This data can be found at: https://rdr.ucl.ac.uk/articles/dataset/Malaria_Prevalence_in_Large_Densely-Populated_Urban_Holoendemic_sub-Saharan_West_Africa_The_Ibadan_1996_to_2017_Dataset/12369137.

## Ethics statement

The studies involving humans were approved by the internationally recognized Ethics Committee at the Institute for Advanced Medical Research and Training (IMRAT) of the College of Medicine, University of Ibadan (COMUI) approved this research on the platform of the Childhood Malaria Research Group (CMRG) within the academic Department of Pediatrics, University of Ibadan, as well as at school and Primary Care centers throughout the city of Ibadan with permit number: UI/EC/10/0130. The studies were conducted in accordance with the local legislation and institutional requirements. Written informed consent for participation was not required from the participants or the participants' legal guardians/next of kin in accordance with the national legislation and institutional requirements.

## Author contributions

TF and DF-R contributed to conception and design of the study. DF-R organized the dataset. TF performed the coding and results collection, wrote the first draft of the manuscript, and wrote the manuscript. TF, DF-R, and SR-G contributed with conceptual and results discussion, manuscript revision, read, and approved the submitted version.
