# Supplementary material for: Specialist hybrid models with asymmetric training for malaria prevalence prediction
Source: Front Public Health. 2023 Sep 21;11:1207624. doi: 10.3389/fpubh.2023.1207624 (PMC10552258; doi:10.3389/fpubh.2023.1207624)
Supplement: Supplementary file 1 [file Data_Sheet_1.PDF]

Specialist hybrid models with asymmetric training for malaria prevalence prediction: Supplementary Material

THOMAS FISHER, SERGIO ROJAS-GALEANO AND DELMIRO FERNANDEZ-REYES

1. MODEL DETAILS

A. Holt-Winters

Holt-Winters models are also called triple exponential smoothing models. These models use weighted average of a increasing numbers of the past observations, with weights given to past observations are proportional to the terms of the geometric or exponential progression. Details are found in [1].

We found that the best parameters (as measure using validation Root Mean Squared Error) for this problem were:

- No trend component is used in the model
- The trend component is damped
- An additive seasonal component is used
- A 12 month seasonal period is used
- Apply the Box-Cox transformation to the data first

B. SARIMAX

SARIMAX is ARIMA with additional seasonal (S) and exogenous (X) components. As this model will later become our classical model of choice we will explain it here for completeness. To start we will explain ARIMA, first proposed in [2].

B.1. ARIMA

Given a time series data X_t where t is an integer index and the X_t are real numbers, an ARMA (p', q) model is given by

$$X_t - \alpha_1 X_{t-1} - \cdots - \alpha_{p'} X_{t-p'} = \varepsilon_t + \theta_1 \varepsilon_{t-1} + \cdots + \theta_q \varepsilon_{t-q} \tag{S1}$$

which we write as

$$\left(1 - \sum_{i=1}^{p'} \alpha_i L^i\right) X_t = \left(1 + \sum_{i=1}^{q} \theta_i L^i\right) \varepsilon_t \tag{S2}$$

where L^i is the lag operator to the ith power which acts as $L^i X_t = X_{t-i}$. Hence an observation at time t is written as a linear combination of previous observations and their associated i.i.d noise values $\varepsilon_j \sim \mathcal{N}(0, \sigma^2)$ for all j.

For an ARIMA model we assume that the lag polynomial $\left(1 - \sum_{i=1}^{p'} \alpha_i L^i\right)$ can be factorised as

$$\left(1 - \sum_{i=1}^{p'} \alpha_i L^i\right) = \left(1 - \sum_{i=1}^{p'-d} \varphi_i L^i\right) (1 - L)^d \tag{S3}$$

and defining $p := p' - d$ we obtain the ARIMA (p, d, q) model

X Variable	MSE	% WT
None (SARIMA)	0.0036	73.68
Host Variables	0.0047	76.30
Weather Variables	0.1285	23.73
Intervention	0.0039	76.32

Table S1. Best (2,0,3)x(2,0,3,12) SARIMAX model prediction MSE and %WT scores for different X (exogenous) variables.

$$\left(1 - \sum_{i=1}^{p} \varphi_i L^i\right) (1-L)^d X_t = \left(1 + \sum_{i=1}^{q} \theta_i L^i\right) \varepsilon_t$$

which we write as

$$\varphi\left(L\right) X_t = \theta\left(L\right) \varepsilon_t \tag{S4}$$

where we have defined

$$\varphi\left(L\right) := \left(1 - \sum_{i=1}^{p} \varphi_i L^i\right) (1-L)^d \text{ and } \theta\left(L\right) := \left(1 + \sum_{i=1}^{q} \theta_i L^i\right)$$

B.2. SARIMA

Introduce the seasonal (S) component, SARIMA uses a season length S and seasonal AR, differenceing and MA orders of P, D, Q respectively. These determine the powers of lag operators in additional polynomials which act multiplicatively:

$$\Phi\left(L^S\right) \varphi\left(L\right) X_t = \Theta\left(L^S\right) \theta\left(L\right) \varepsilon_t \tag{S5}$$

where the Φ and Θ polynomials are

$$\Phi\left(L^S\right) := \left(1 - \sum_{i=1}^{P} \Phi_i L^{Si}\right) (1-L^S)^D \text{ and } \Theta\left(L^S\right) := \left(1 - \sum_{i=1}^{Q} \Theta_i L^{Si}\right)$$

B.3. SARIMAX

To include an exogenous variable Z_t we simply allow for the addition of a linear term:

$$\Phi\left(L^S\right) \varphi\left(L\right) X_t = \Theta\left(L^S\right) \theta\left(L\right) \varepsilon_t + \beta Z_t \tag{S6}$$

Exogenous variables are determined outside the model and are imposed on the model. For example we could choose Z_t to have values correspond to different antimalarial interventions in place.

Results from using SARIMAX with different exogenous variables are shown in Table S1. We also include a baseline of the SARIMA model, which does not have any exogenous variables.

We see that the forecasting ability for the SARIMAX models using host variables and intervention as exogenous variables and the SARIMA model without an exogenous variables obtain good validation set MSE and MPET performance.

C. Elastic Net

Originally proposed by [3], the Elastic Net works just like linear regression but with control of the L1 and L2 norms of the parameter vector to minimise the objective function

$$\mathcal{O}_{\mathrm{EN}}(w) = \|y - Xw\|^2 + \alpha\lambda\|w\|_1 + \alpha(1-\lambda)\|w\|^2 \tag{S7}$$

where $\|a\|_1 := \sum_d |a_d|$ is the L1-norm and $\|\cdot\|$ is the usual L2-norm. The hyper parameters α and λ control sparsity and the grouping effect.

This method linearly combines the L1 and L2 penalties of the well-known lasso and ridge methods and has the advantage that the solution is regularised and avoids overfitting.

We used a grid search with 10-fold cross validation to obtain the best hyper parameter values of $\alpha = 0.0005$ and $\lambda = 0.075$.

The study by [4] on this same dataset fount that Elastic Net was the best model for prevalence prediction. Hence we can use this as a benchmark for the performance of the models along with the simple averages.

D. Random Forest

Random Forest is a boostrapping and bagging method to combine a large number of decision trees in an ensemble. The fundamental concept behind random forest is a simple but powerful wisdom of crowds.

The individual decision trees are trained on randomly sampled subsets of features and training data points drawn with replacement, which ensure that the individual trees are not correlated. Predictions are then made by averaging the predictions from the set of individual trees. This method reduces variance of the estimator and decrease issues of overfitting common to standard decision trees. A large number of relatively uncorrelated trees operating as a committee will outperform any of the individual constituent themselves.

We use 100 different individual treeestimators trained using MSE on boostrapped data.

Ensembling reduces the variance and increases the quality of the estimate. We set no maximum depth to the tree and so nodes are expanded until all leaves are pure or until all leaves contain fewer than 2 samples.

E. Support Vector Regression

The method was first proposed by [5]. In this approach we fit a linear function $f(x; w, b) = \langle w, x\rangle + b$ to minimise the objective function with slack variables ξ_i

$$\frac{1}{2}\|w\|^2 + C\sum_{i=1}^{N}(\xi_i^+ + \xi_i^-) \tag{S8}$$

subject to

$$y_i - \langle w, x_i\rangle - b \leq \epsilon + \xi_i^+ \tag{S9}$$

$$\langle w, x_i\rangle + b - y_i \leq \epsilon + \xi_i^- \tag{S10}$$

$$\xi_i^+, \xi_i^- \geq 0 \tag{S11}$$

such that any deviations from the observed targets are less than ϵ (defining a margin around the true values) do not get penalised. The aim is to maximize the margin for a lower generalization error. The final solution produces in a subset of training points that define the margin which are referred to as support vectors.

The constant C acts as a regularisation parameter, and can be interpreted as how flexible the model is and choosing where the model lies in terms of bias-variance tradeoff.

This can be shown to have a solution which evaluates at a test point x as

$$y(x) = \sum_{i=1}^{N}\alpha^*\langle x_i, x\rangle + b \tag{S12}$$

for some α^* as we would expect by the Representer Theorem [6]. This is trivially extended to using feature maps and kernels rather than just standard inner products by using the solution

$$y(x) = \sum_{i=1}^{N} \alpha^* K(x_i, x) + b \tag{S13}$$

We used a radial square exponential basis function (RBF) kernel

$$K(x, x') := \exp(-\gamma \|x - x'\|^2) \tag{S14}$$

where the length scale γ controls the "wiggliness" of the function.

We performed a random search 10,000 times over the hyperparameter space in order to obtain the best values of $C = 24, \epsilon = 0.023, \gamma = 0.0022$

F. Artificial Neural Network

Originally conceptualised by [7], with the training methods of backpropagation originating in [8], artificial neural networks (NNs) are a flexible family of models that have been used successfully in a hugely varied collection of real-world applications. The model is composed of a number of layers of nodes which perform linear operations on its inputs (activations of the nodes from the previous layer) and then apply a nonlinear function and give this activation as input for the nodes in the next layer.

The activation of the jth neuron in the kth layer is obtained from the activations in the $(k-1)$th by the equation

$$a_j^k = \sigma\left(\sum_{i=1} w_{ji}^k a_i^{k-1} + b_j^k\right) \tag{S15}$$

where σ here is a nonlinear activation function and w_{ji}^k is the weight for the connection from the ith neuron in the $(k-1)$th layer to the jth neuron in the kth layer and b_j^k is the bias of the jth neuron in the kth layer. Further details can be found in [9]. As was shown by [10] these neural networks universal - they are able to approximate any function arbitrarily closely given enough training data.

We use ReLU as our activation function in this study. The network is trained using using the ADAM adaptive backpropagation optimiser [11].

Keeping in mind the rules of thumb from [12] that hidden layers should not be more than twice as large as the input layer and that the total number of hidden nodes should not be three times the size of the input data, we performed a gridsearch over networks of three hidden layers with up to 10 neurons in each layer. The optimal model was found to have a 6-8-6 architecture.

G. LSTMs

G.1. Vanilla LSTMs

The equations for and LSTM cell are

$$f_t = \sigma\left(W_{fh} h_{t-1} + W_{fx} x_t + b_f\right) \tag{S16}$$

$$i_t = \sigma\left(W_{ih} h_{t-1} + W_{ix} x_t + b_i\right) \tag{S17}$$

$$\tilde{c}_t = \tanh\left(W_{ch} h_{t-1} + W_{cx} x_t + b_c\right) \tag{S18}$$

$$c_t = f_t \cdot c_{t-1} + i_t \cdot \tilde{c}_t \tag{S19}$$

$$o_t = \sigma\left(W_{\alpha h} h_{t-1} + W_{ax} x_t + b_o\right) \tag{S20}$$

$$h_t = o_t \cdot \tanh\left(c_t\right) \tag{S21}$$

where h_t is the recurrent information, c_t is the cell state,f_t is the forget gate, i_t is the input gate, o_t is the output gate and $\tilde{c}_t$ is intermediate values calculated to update the cell state. The Ws are weights and the bs are biases. Here $\cdot$ denotes pointwise multiplication of vectors. σ is the sigmoid function.

The forget gate architecture allows the model to specify how much of the cell state should be kept. When the value of the forget gate is 1, it keeps all of this information and a value of 0 means it gets rid of it all. When updating the cell state, the input gate can decide what new information can be stored in the cell state, and the output gate decides what information can be output based on the cell state. This architecture allows LSTMs to handle long term dependencies much better than RNNs.

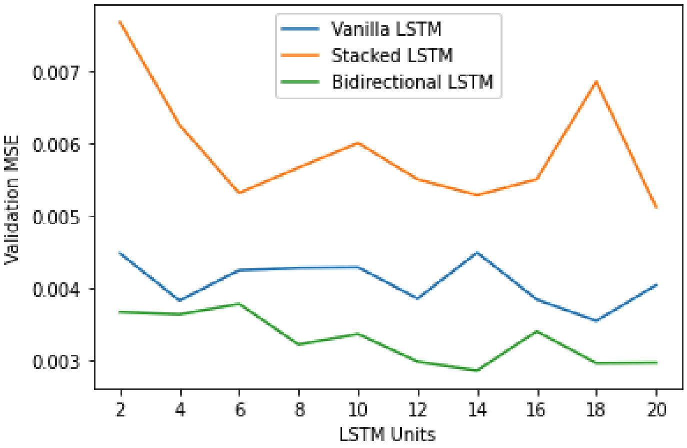

Fig. S1. Validation MSEs for LSTMs of different sizes and of different architecture types.

G.2. Stacked LSTMs

Stacking LSTMs involves connecting layers of units on top of each other. The output of cells in one layer are the input to cells in the next layer. The intuition is that higher LSTM layers may be able to capture more abstract concepts in the sequences being investigated. Here we investigated only a singly stacked, double-decker architecture.

G.3. Bidirectional LSTMs

Bidirectional LSTMs connect two units reading the information in the opposite directions to the same output. When looking at timeseries data this allows them to preserve information from both past and future.

In order to allow for fair comparison between Bidirectional and Vanilla LSTMs we counted every bidirectional unit as two vanilla LSTM units. This ensures that the models being compared have the same number of parameters.

Figure S1 shows that the Bidirectional LSTMs are the best performing models by validation MSE performance for this problem. Hence they will be used as the LSTM model architecture of choice for our work.

2. PREVALENCE TRANSFORMATION

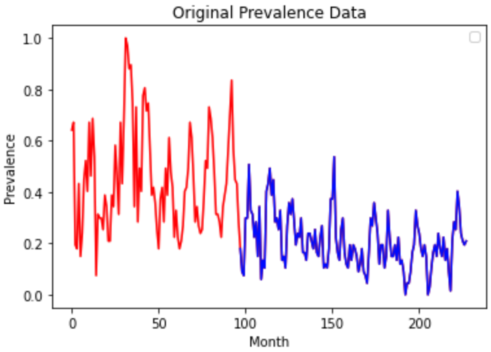

Fig. S2. Prevalence before transformation

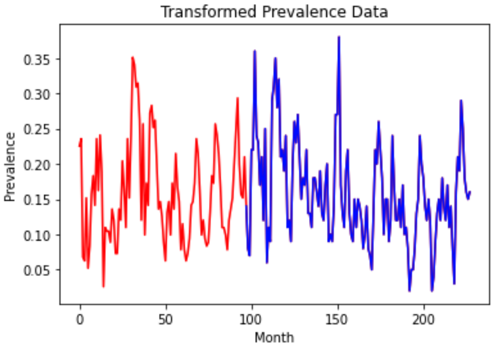

Fig. S3. Prevalence after transformation

In a data-sparse setting like this we must make every effort to ensure that our models are trained on the the largest datasets possible that are representative of the real-world phenomena.

Let μ, μ' and σ, σ' be the means and standard deviations of the prevalence samples before and after the introduction IPT-Preg as an intervention respectively. These samples are shown on the left in blue and on the right in red in Figure S2.

For each month t before the introduction of IPT-Preg we transform the prevalence $y(t)$ as

$$y_{\text{trans}}(t) := \frac{\sigma'}{\sigma} \times (y(t) - \mu) + \mu' \quad \text{(S22)}$$

This process ensures that the standard deviation and mean of the transformed prevalence is the same as the prevalence for the Post data when the IPT-Preg intervention is in place, resulting in the transformed prevalence shown in red on the left of Figure S3. Note the y-axis ranges of the two plots. Note that the data after the introduction of IPT-Preg prevalence shown in blue remains the same as the data is not altered in any way. Ideally all of the collected data would be under the influence of the same interventions as the test period. By carrying out this process we synthesise what the Pre data might have looked like if all of the Post interventions were in place during this period.

3. MODEL PERFORMANCE USING TRANSFORMED AND NON-TRANSFORMED PREVALENCE

Table S2 shows the improved model test performance when trained on the transformed prevalence data.

Model	Original Data		Trans. Data	
	MSE	%WT	MSE	%WT
Average	0.032	14.7	0.0041	77.1
Monthly Average	0.044	20.0	0.0065	62.9
HW	0.0068	54.0	0.0068	54.3
SARIMAX	0.0057	76.3	0.0058	76.4
EN	0.0085	60.0	0.0028	77.1
RF	0.0060	65.7	0.0034	80.0
SVR	0.0062	62.9	0.0032	71.4
NN	0.031	31.4	0.0027	84.7
BiLSTM	0.012	71.4	0.0024	85.3

Table S2. Test set performance of models trained on the original and transformed datasets as explained in Section 2. For the neural models requiring initialisaion these values were obtained by averaging to

REFERENCES

1. C. C. Holt, “Forecasting seasonals and trends by exponentially weighted moving averages,” Int. J. Forecast. **20**, 5–10 (2004).
2. P. Whittle, *Hypothesis Testing in Time Series Analysis*, Statistics / Uppsala universitet (Almqvist & Wiksells boktr., 1951).
3. H. Zou and T. Hastie, “Regularization and variable selection via the elastic net,” J. Royal Stat. Soc. Ser. B: Stat. Methodol. **67**, 301–320 (2005).
4. B. J. Brown, A. A. Przybylski, P. Manescu, F. Caccioli, G. Oyinloye, M. Elmi, M. J. Shaw, V. Pawar, R. Claveau, J. Shawe-Taylor, M. A. Srinivasan, N. K. Afolabi, A. E. Orimadegun, W. A. Ajetunmobi, F. Akinkunmi, O. Kowobari, K. Osinusi, F. O. Akinbami, S. Omokhodion, W. A. Shokunbi, I. Lagunju, O. Sodeinde, and D. Fernandez-Reyes, “Data-Driven Malaria Prevalence Prediction in Large Densely-Populated Urban Holoendemic sub-Saharan West Africa: Harnessing Machine Learning Approaches and 22-years of Prospectively Collected Data,” Tech. rep. (2019).
5. H. Drucker, C. J. Surges, L. Kaufman, A. Smola, and V. Vapnik, “Support vector regression machines,” in *Advances in Neural Information Processing Systems*, (1997), pp. 155–161.

6. B. Schölkopf, R. Herbrich, and A. J. Smola, "A generalized representer theorem," in *Lecture Notes in Computer Science (including subseries Lecture Notes in Artificial Intelligence and Lecture Notes in Bioinformatics)*, vol. 2111 (2001), pp. 416–426.
7. W. S. McCulloch and W. Pitts, "A logical calculus of the ideas immanent in nervous activity," The Bull. Math. Biophys. **5**, 115–133 (1943).
8. P. Werbos, *Beyond Regression: New Tools for Prediction and Analysis in the Behavioral Sciences* (Harvard University, 1975).
9. A. Sherstinsky, "Fundamentals of Recurrent Neural Network (RNN) and Long Short-Term Memory (LSTM) network," Phys. D: Nonlinear Phenom. **404** (2020).
10. B. Csáji, "Approximation with artificial neural networks," MSc. thesis p. 45 (2001).
11. D. P. Kingma and J. L. Ba, "Adam: A method for stochastic optimization," in *3rd International Conference on Learning Representations, ICLR 2015 - Conference Track Proceedings*, (2015).
12. M. Berry and G. Linoff, *Data mining techniques: for marketing, sales, and customer relationship management* (2004).
